# Pattern of retinal morphological and functional decay in a light-inducible, rhodopsin mutant mouse

**DOI:** 10.1038/s41598-017-06045-x

**Published:** 2017-07-18

**Authors:** Claudia Gargini, Elena Novelli, Ilaria Piano, Martina Biagioni, Enrica Strettoi

**Affiliations:** 1 Department of Pharmacy, University of Pisa, Pisa, Italy; 2 CNR Neuroscience institute, Pisa, Italy

## Abstract

Hallmarks of Retinitis Pigmentosa (RP), a family of genetic diseases, are a typical rod-cone-degeneration with initial night blindness and loss of peripheral vision, followed by decreased daylight sight and progressive visual acuity loss up to legal blindness. Great heterogeneity in nature and function of mutated genes, variety of mutations for each of them, variability in phenotypic appearance and transmission modality contribute to make RP a still incurable disease. Translational research relies on appropriate animal models mimicking the genetic and phenotypic diversity of the human pathology. Here, we provide a systematic, morphological and functional analysis of Rho^Tvrm4^/Rho^+^ rhodopsin mutant mice, originally described in 2010 and portraying several features of common forms of autosomal dominant RP caused by gain-of-function mutations. These mice undergo photoreceptor degeneration only when exposed briefly to strong, white light and allow controlled timing of induction of rod and cone death, which therefore can be elicited in adult animals, as observed in human RP. The option to control severity and retinal extent of the phenotype by regulating intensity and duration of the inducing light opens possibilities to exploit this model for multiple experimental purposes. Altogether, the unique features of this mutant make it an excellent resource for retinal degeneration research.

## Introduction

A large variety of experimental approaches aimed at sight restoration or prevention of vision loss caused by retinal degeneration have been recently proposed. Irreversible photoreceptor diseases can arise from hundreds different gene mutations (RetNet, the Retinal Information Network, at http://www.sph.uth.tmc.edu/RetNet/) and visually impaired people doomed to blindness are estimated in over 20 million worldwide. Among them, individuals suffering from Retinitis Pigmentosa (RP) share a phenotypically broad correspondence characterized by a typical rod-cone-degeneration with initial night blindness and loss of peripheral vision. Subsequently also daylight vision and visual acuity are impaired, ending up with legal blindness. RP is a family of disorders typically caused by a single mutation in any one of numerous, often functionally distant genes, 60 of which have been identified so far. They may encode for a variety of proteins and among them: visual cycle or phototransduction enzymes, ciliary traffic proteins, transport and cytoskeleton components, ion channels, transcription factors, nuclear receptors^1^. Most of the genes involved in RP are specific for photoreceptors, or the retinal pigment epithelium (RPE), or the retina; RP mutations can be transmitted according to autosomal dominant, recessive, X-linked and mitochondrial patterns of inheritance. Thus, defining RP as a highly genetic heterogeneous disease is justified by the large number of underlying mutations, the diverse functions of mutated genes and the variable modality of inheritance, notwithstanding the occurrence of a fairly homogeneous phenotype.

Some 40% of Retinitis Pigmentosa cases display autosomal dominant inheritance, and 25–30% of these are attributable to mutations in *RHO*, the gene coding for rhodopsin, the light sensitive protein of rod photoreceptors. A biochemical classification introduced in the 90thies^2, 3^ identifies two classes of mutations (I and II) of *RHO*. Class I mutations mostly occur near the C-terminal of the protein, which still retains the ability to bind to 11-*cis*-retinal and to form a functional chromophore but may not be properly transported to the outer segment and shows constitutive activation or increased transducin activation^4^. The more common class II mutations occur in the transmembrane or cytoplasmic domains of the protein, resulting in rhodopsin misfolding, retention in the endoplasmic reticulum and failure to bind to 11-cis-retinal.

Dominant mutations might be associated to a gain-of-function of the endproduct, as shown for the P23H class II mutation, where rhodopsin retention in the ER is followed by a continuous unfolded protein response (UPR), inhibition of the proteasome and aggregation in high molecular weight oligomers, ultimately forming toxic inclusion bodies^5, 6^. Such mutations are particularly challenging for gene therapy approaches, as they require suppression of the expression of the native gene and gene augmentation at the same time^7^. Clearly, rhodopsin stability and efficiency tolerates only few aminoacid changes, as reflected in the finding that the protein shares more than 95% sequence identity even in different species, like mice and humans. Rhodopsin expression, synthesis, folding, translocation to the outer segments of rods and turnover are highly regulated processes, whose impairment can easily lead to cell death, also considering that the photopigment contributes some 90% of the whole protein content of outer segment membranes.

Adequate animal models for *RHO* mutations are of key importance for understanding the complex link between primary genetic defect and cell death, and for developing appropriate approaches for vision preservation in humans with identical or similar mutations. Yet, available *RHO* models are few compared to the number of known mutations in the rhodopsin gene: P23H mice and rats mimic the most common human mutation of *RHO* and have been exploited extensively; however, some strains manifest their phenotypes relatively late in life and with a too slow progression for efficient testing of experimental treatments; moreover, P23H mice are transgenics^8^ like T17M, P347S^9^ and S334X mice (reviewed in: ref. 10), and produce mutant as well as wild type rhodopsin, believed to be toxic at higher than physiological levels. Other mutants, such as the C185R (or R3) mouse^11^ undergo a severe retinal degeneration starting between 2 and 3 weeks of life, during late retinal development, thus combining the effects of photoreceptor death with those of an improper retinal maturation. This is a discrepancy with respect to typical human RP, which usually occurs in fully developed retina.

Here, we provide the secondary characterization of retinal phenotype of a mouse strain (Rho^Tvrm4^, from now on referred to as “Tvrm4”) originally described by^12^, and constituting a model of autosomal dominant RP where a missense mutation of *RHO* changes amino acid 307, isoleucine (ATC), to asparagine (AAC). Ile-307 is in the 7th transmembrane region of rhodopsin and is conserved across species, including humans. Peculiar features of Tvrm4 mice are that a) being generated by mutagenesis, they are not transgenics and have normal levels of rhodopsin; and b) they do not show retinal abnormalities if grown in normal ambient light but undergo RP-like retinal degeneration when exposed briefly to strong, white light, which is otherwise innocuous for wild type (wt) controls.

The following is an analytical description of the main phases of photoreceptor morphological and functional degeneration induced in adult Tvrm4 mutants, illustrating the advantageous possibility of modulating the spatial and temporal severity of the phenotype as a function of inducing light intensity and duration.

## Results

### Retinal morphology in Tvrm4 mice after phenotype induction

**Figure 1:**
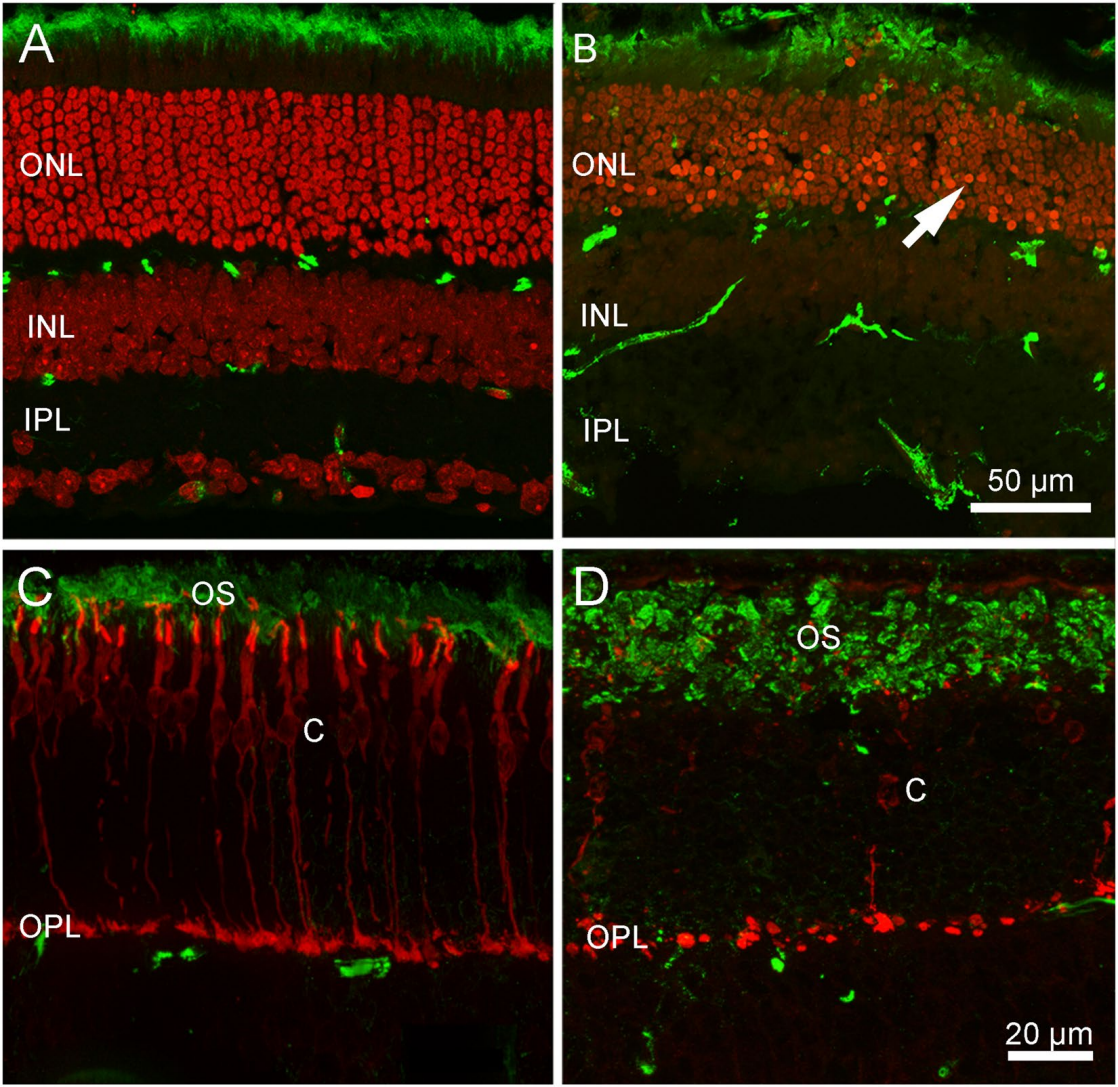
Early effects of bright light exposure. (**A**,**C**) Control retinas from Ht Tvrm4 mice before bright light exposure; (**B**,**D**) Central retinal sections from Ht Tvrm4 littermates exposed to 12,000 lux for 1 min and examined after 48 hrs. (**A**,**B**) Green signal: ICCH for the light sensitive channel of rods; red: ethidium nuclear counterstaining. Note the thinner ONL and the abundance of pycnotic nuclei in (**B**) (arrow). (**C**,**D**) cone arrestin (red) and rhodopsin (green). Clustering of rhodopsin in the outer segments of rods (OS) is evident in (**D**) where cones are also poorly stained and at a lower density.

The retina of young adult Tvrm4 mice (both hetero-, Ht, and homozygous for the H307N mutation) maintained in normal ambient light is morphologically undistinguishable from that of wild type (wt, C57Bl6/J) littermates and shows no signs of abnormality in general organization and laminar structure (Fig. 1A,C). A normal retinal morphology is also found in Ht Tvrm4 mutants (used throughout the study) exposed to 12,000 lux light pulses of various durations (i.e. 1, 2, 3, 4 or 5 minutes) but not treated with atropine for pupil dilation (data not shown). Hence, non-induced Tvrm4 mutants bear no major phenotypical distinction from wt littermates, at least during the first 6 months of life, the oldest age examined here. This confirms previous data from^12^.

**Figure 2:**
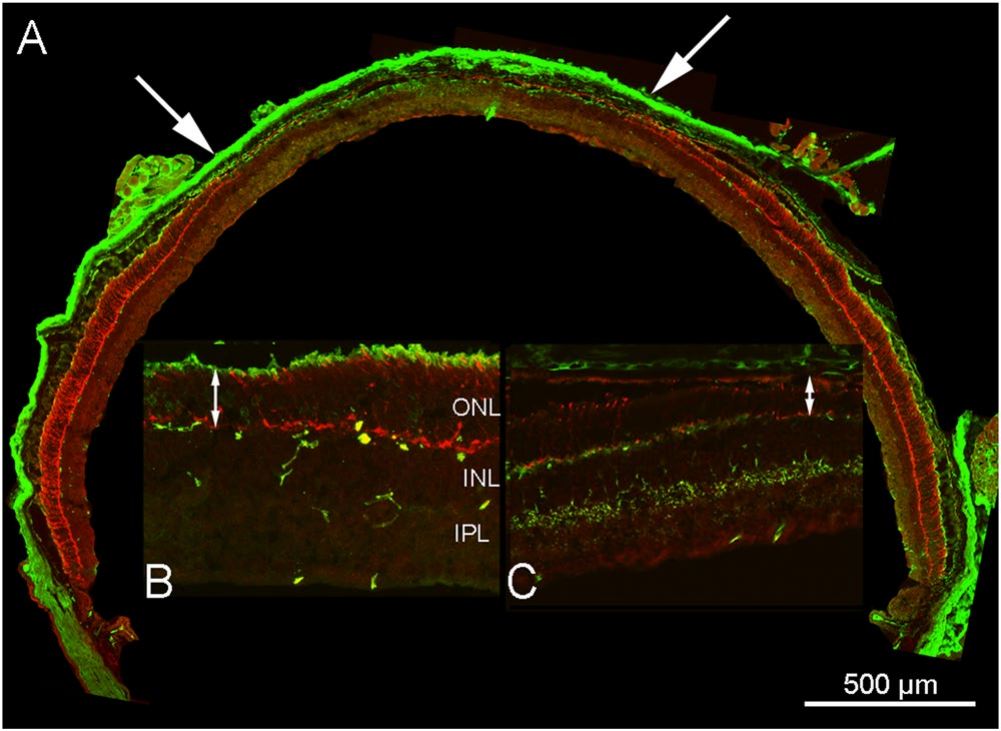
Retinal morphology 7 days post induction. Vertical section of a complete eye cup from a Tvrm4 mouse exposed to 12,000 lux for 1 min and examined after 7 days. Red: cone arrestin; green: light sensitive channel staining. The central area devoid of photoreceptors (between the two arrows in (A) is highlighted by the drastic reduction of both rod and cone markers, totally undetectable. (B and C) higher magnification images of a preparation similar to A. (B) same staining as in A, showing the decreasing thickness of the ONL (double arrow). (C) cone arrestin (red) and synaptotagmin staining (green) show cone bipolar cells whose axons form a thick plexus in the inner plexiform layer (IPL; on the contrary, the ONL is progressively thinning (double arrows) and cones (red) become gradually shorter.

**Figure 3:**
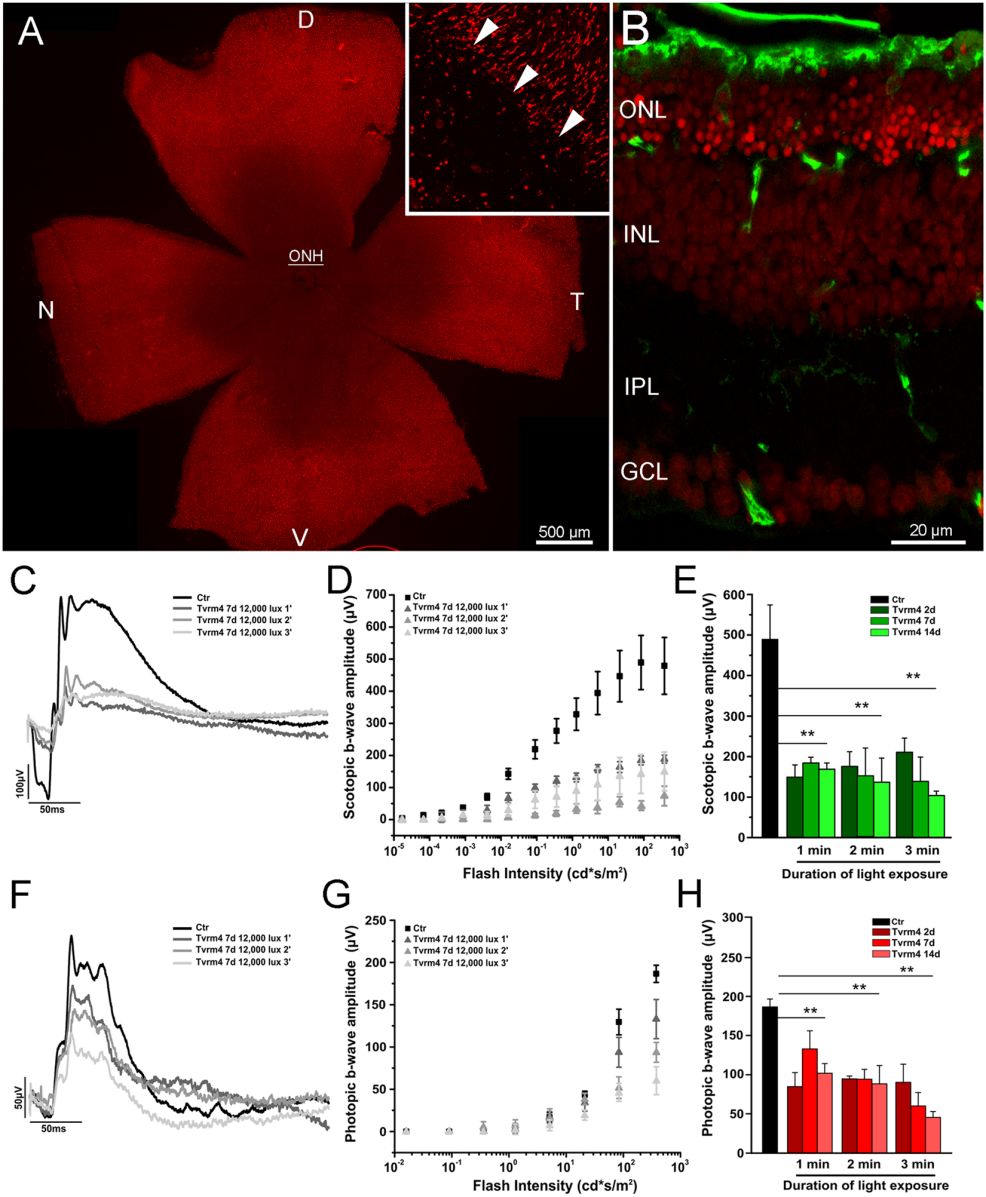
Retinal phenotype 7 days post induction: structure and functional correlation. (**A**) Retinal whole mount from a Tvrm4 mouse exposed to 12,000 lux for 1 min and examined after 7 days. ICCH for short- and medium-wavelength cone opsins (red signal) shows a large area flanking the optic nerve head (ONH) and devoid of cones. Inset: transition margin (arrowheads) between the cone-free central area and a more peripheral zone with still intact cone outer segments. In this and all the whole mount images; D, V, N, T: dorsal, ventral, nasal and temporal retinal quadrants. (**B**) Vertical section from a retinal sample as that shown in A, at intermediate eccentricity between the ONH and the retinal peripheral edge. Green: rhodopsin staining. Red: ethidium staining. The ONL is only 6 rows thick, as opposed to the typical 10–12 rows in the intact retina. Remaining rods have shorter outer segments. Pycnotic nuclei in the innermost part of the ONL indicate active photoreceptor DNA condensation. Representative scotopic (**C**) and photopic (**F**) flash ERG responses recorded from 3 groups of Ht Tvrm4 mice before (black trace) and after (gray traces) exposure to 12,000 lux for 1, 2 or 3 minutes. ERGs were recorded after 7 days. (**C,F**) examples of ERG in response to the brightest flash. (**D,G**): scotopic and photopic b-wave amplitudes as a function of flash intensity in the three experimental groups. (**E,H**) histograms of maximum b-wave amplitude in response to the brightest flash in animals of the three experimental groups. Data were from n = 6 animals/condition. Bars are mean ± SE; **p < 0.01 one-way ANOVA. Scotopic brightest flash: 83.7 cd * s/m^2^; photopic brightest flash: background 30 cd/m^2^ + superimposed flash 377 cd * s/m^2^.

Exposure of Tvrm4 mice with dilated pupils to a light pulse of 1′ and 12,000 lux intensity causes a brisk wave of photoreceptor degeneration detectable in 48 hrs. This surge in photoreceptor degeneration is illustrated by the immunostained retinal sections of Fig. 1B,D. Numerous pycnotic nuclei, brightly stained with DNA-binding molecules, are evident in the outer nuclear layer (ONL) (Fig. 1B). More detailed microscopic analysis shows that some of these nuclei have the typical small size and fragmented appearance of apoptotic bodies. Rhodopsin immunolabeling shows swollen and broken rod outer segments with clustered staining product, while cellular debris can be observed in the subretinal space (Fig. 1D). Cones are more scattered than normal and show abnormal morphologies with shortened outer segments and distorted profiles (Fig. 1B). One week post induction, the central retina exhibits a clear pattern of degeneration that involves both rods and cones, as one may appreciate from vertical sections and in whole mount preparations as well (Figs 2, 3). The irradiated zone, highlighted by a faint cone-opsin staining, has an average radius of 1–1.3 mm from the optic nerve head, covering a surface of approximately 5.3 mm^2^, or 32–35% of the retina, which has an average area of 16.0 mm^2^. At times, the irradiated spot has an irregular shape and is less extended on the temporal quadrant. Inside this visible “black hole”, the ONL thickness has decreased from the normal 10–12 rows of nuclei to approximately 6 rows. Remaining rods have shortened and irregular outer segments (Fig. 2A,B). A transition zone with a thicker ONL and gradually more intact cones is clearly visible in equatorial sections of eye cups (Fig. 2A) and in whole mount preparations (Fig. 3A). It is seen that the pattern of ONL degeneration is maximum in the central, directly irradiated retinal region. Cone arrestin staining demonstrates distorted cone profiles, enlarged inner segments and shortened or virtually absent outer segments (Figs 2A and 3A).

Immunocytochemistry for a cone bipolar cell marker (synaptotagmin) highlights the discrepancy between the morphology of the seriously degenerated outer retina, and that of the inner layers, where the axonal arbors of cone bipolar cells outline a still intact lamination (Fig. 2C). In whole mount preparations, the transition between irradiated and intact zone can be appreciated in the cone opsin staining images, showing that cone outer segments get shorter and at low density while progressing toward the central retina (Fig. 3, inset). Noticeably, exposure of wt mice to the same intensity of light described above, even for longer times (i.e. 12,000 lux for 1, 2, 3, 4 or 5 minutes) does not result in any sign of photoreceptor or retinal degeneration, as assessed by morphological studies (Fig. 3B, Suppl. Figs 1 and 2) and ERG recordings. Therefore, the strong light *per se* is not sufficient to evoke a classical retinal light-damage; rather, a combination of Tvrm4 mutation and light is necessary. Light damage studies have shown higher susceptibility of both very young^13^ and aging^14^ rodents to retinal degeneration; however, we did not observe any difference in phenotype manifestation among the Tvrm4 mice we studied, which were anyway all in the range of young adulthood (i.e. between 2 and 5 months of age).

### Retinal physiology in Tvrm4 mice after phenotype induction

To determine the functional correlates of the morphological findings described above, we performed an extensive retinal functional assay on Ht Tvrm4 mice subjected to three protocols of light-induction (12,000 lux for 1, 2, 3 min) and in which we recorded the flash electroretinogram (ERG) in scotopic and photopic conditions at different times (2, 7, 14, 30 days) from phenotype induction.

We confirmed that before exposure to bright light pulses, the ERG of Tvrm4 mutants is comparable to that of age-matched wt animals (data not shown). However, a drastic reduction of both scotopic (Fig. 3C–E) and photopic (Fig. 3F–H) responses is visible in Tvrm4 mice exposed for 1 min to 12,000 lux. Figure 3E shows that a significant (p < 0.01, ANOVA) impairment of the scotopic ERG arises as early as 2 days after light-induction for all the light exposure protocols. The same result is obtained for the photopic ERG (p < 0.01, ANOVA, Fig. 3H).

The scotopic ERG suppression is similar for all induction protocols used and at all recorded times. The reduction of the scotopic and photopic b-wave depends, however, on the time of exposure to the light source, so that for the longest exposure (3 min) the response is more reduced at longer times from the induction (Fig. 3E,H).

**Figure 4:**
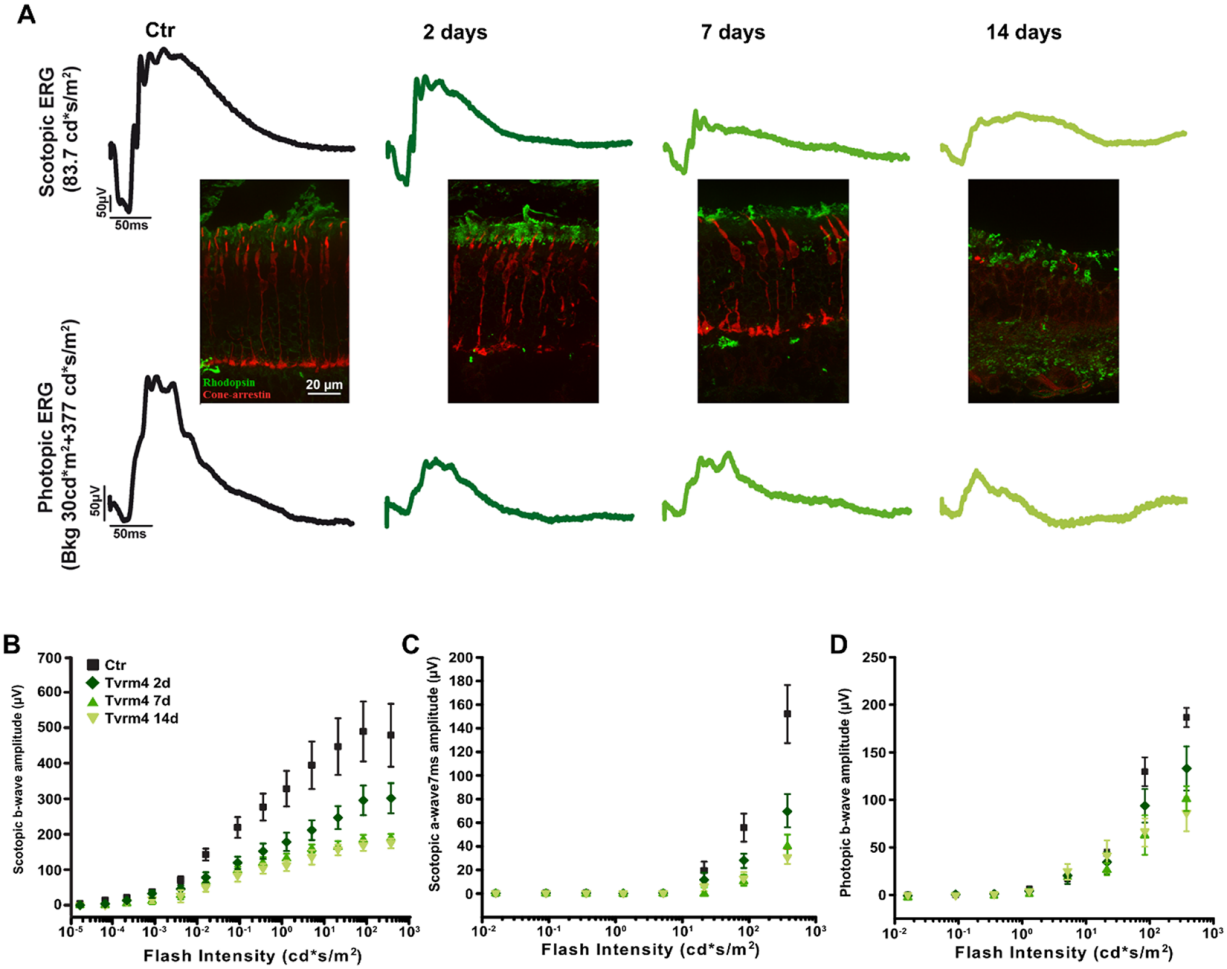
Spatial and temporal progression of phenotype. Correlation of ERG decay and morphological impairment of the central retina of Tvrm4 mice exposed to 1 minute, 12,000 lux conditioning light and examined after 2, 7, and 14 days. (**A**) Upper traces: example of scotopic ERG. Bottom traces: photopic ERG. Immunofluorescence staining insets show the progressive loss of rhodopsin immunoreactivity and disappearance of cones from the central retina in vertical sections (green: rhodopsin staining; red: cone-arrestin). (**B** and **C**) scotopic b-wave and a- wave amplitudes as a function of flash intensity in control mice and in Tvrm4 mice exposed as above. (**D**) photopic b-wave amplitudes. Note that before phenotype induction (pre-illumination condition, Ctr) ERG traces and retinal morphology of Tvrm4 are normal. Data collected from n = 6 animals for each experimental condition. Each point represents the mean ± SE.

Two days after induction, both scotopic and photopic ERG responses start to decline compared to control animals (Fig. 4). After 7–14 days the reduction of the scotopic a- and b-waves tops about 70% (Fig. 4B,C). The photopic ERG is reduced as well but the suppression reaches a peak after two days and remains stable thereafter (Fig. 4D). Conceivably, 1 min light induction results into a partial loss of cones rather than in the massive degeneration of these cells.

Correspondingly, degeneration of the ONL in the central retina area of Tvrm4 mice exposed for 1 min to 12,000 lux is extensive 2 weeks after exposure, when outer segments of rods are greatly decreased in length and density and only few cones can be stained (Fig. 4A). Residual cones are evidently undersized and show abnormal shapes. The ONL degeneration progression remains confined to the retinal central zone, inside the area exposed to the inducing light. Measuring retinal vertical sections from Tvrm4 mice exposed for 1′ at 12000 Lux and harvested at various times, we found that in the spot of maximum photoreceptor degeneration, adjacent to the optic nerve head, the average number of ONL rows (and average of 12 in the retina of unexposed mice) was respectively 8.3 ± 0.3 at 48 hr; 3.3 ± 0.4 at 7 days; 1.2 ± 0.3 at 14 days and 0.3 ± 0.3 after 30 days. Thus, photoreceptor degeneration rate is maximum during the first week post-exposure. Similarly, the central damaged area covered 26% of the retinal surface 48 hr post exposure, and 43%, 32% and 20% respectively after 7, 14 and 30 days (n = 3 retinas per time group measured).

**Figure 5:**
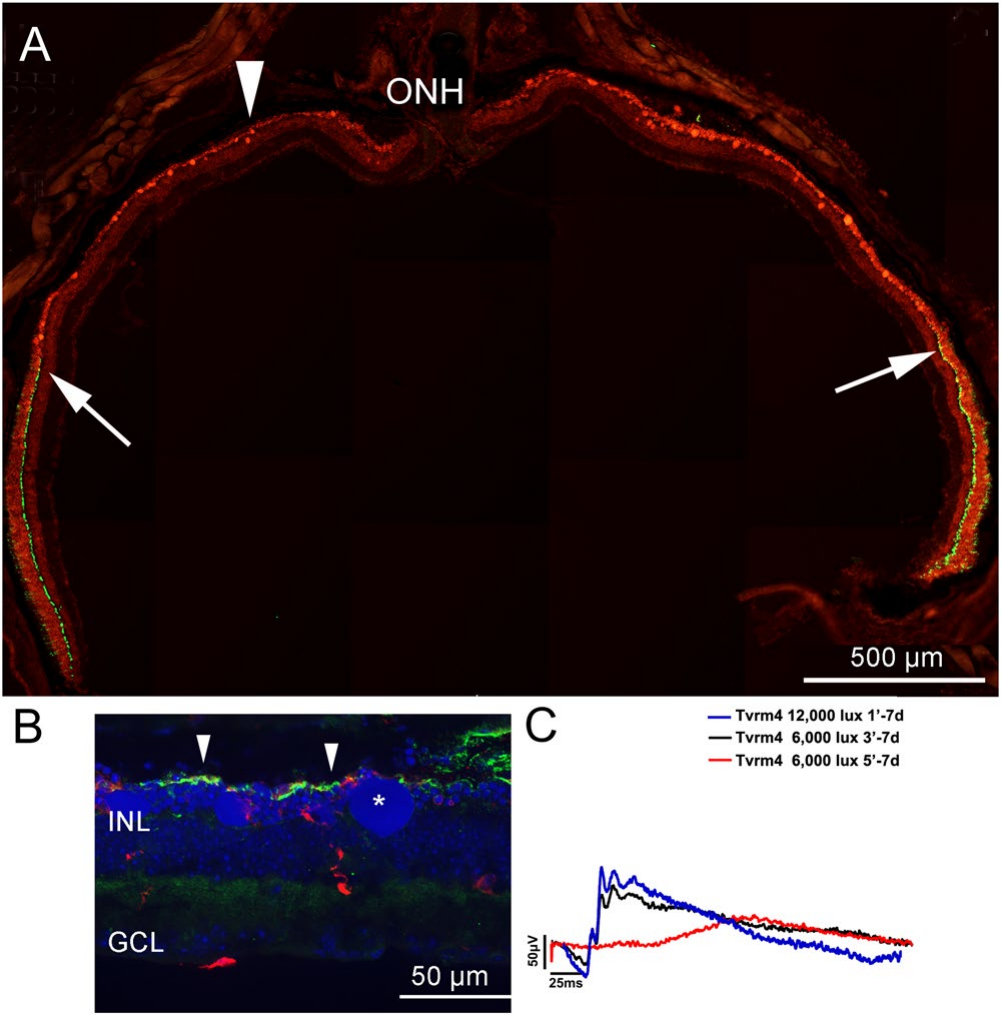
Effects of prolonged time exposure on retinal degeneration. (**A**) Equatorial retinal section obtained 7 days after 5 min exposure to 6,000 lux. (**A**) Cone arrestin (green) and ethidium staining (red) show that a large fraction of the retinal surface has undergone major photoreceptor loss. The ONL is reduced to 3–4 rows across approximately 80% of the retinal extension; cones are appear intact only close to the retinal margins, where the ONL has a normal thickness (arrows). ONH: optic nerve head. (**B**) High magnification of the arrowhead-indicated zone in (**A**). Rhodopsin staining (green) shows extreme reduction of rod outer segments. In this central area, cones (stained with arrestin, red) are rare and show aberrant morphologies. Hoechst DNA staining (blue) shows large apoptotic bodies in the outer retina (asterisk). This preparation conveniently recapitulates in space the degeneration progression typical of Tvrm4 mutants. (**C**) ERG traces obtained 7 days after exposure to inducing lights of different brightness and duration. The larger decrement of the retinal response is observed when exposure length increases from 3 to 5 minutes. Scotopic brightest flash: 83.7 cd*s/m^2^.

Exposure of Tvrm4 mice to the same luminance for increasingly longer times (i.e. 12,000 lux for 2 and 3 minutes, respectively) results in affected areas of larger extension and covering the central 38–40% of the retinal surface, as measured using photoreceptor or DNA staining methods. The corresponding pattern of degeneration is morphologically similar to the one described above (i.e. a rod-cone degeneration with center-to-periphery gradient) but more rapid, with disappearance of the ONL in the central retina achieved in less than 7 days with 3 min illumination. As previously shown by^12^ exposures longer than 1 min cause the formation of large DNA aggregates in the ONL, indicative of the simultaneous degeneration of a large fraction of photoreceptors with consequent condensation of multiple degenerating nuclei, visible in Fig. 5.

Interestingly, we found that longer time exposures at a lower luminance (i.e. 5 min at 6,000 lux) induce a pattern of degeneration covering almost 75% of all the retinal surface and sparing only the far end of the retinal periphery. With this induction protocol, most of the ONL is reduced to 3–4 rows of nuclei one week after light exposure and the photoreceptor degeneration pattern occurs simultaneously over most of the retinal surface. This paradigm of exposure that causes a general photoreceptor decrement and an abrupt decay of scotopic ERG (Fig. 5C) could be employed to obtain synchronous removal of photoreceptors from the whole retina, to study generalized consequences on inner neurons of second and higher order (i.e. genesis of oscillatory networks in the IPL^15, 16^; onset of abnormal discharge properties in ganglion cells^17^).

Because of an advantageous correspondence between morphological and functional observations and a relatively mild pattern of retinal degeneration, we chose to further extend our analysis using a 12,000 lux, 1 min exposure inducing protocol. The induced, gradual paradigm of phenotypic manifestation seems particularly useful for future rescue studies of photoreceptor degenerations (i.e. based on gene therapy or on pharmacological approaches), although one should be aware of the fact that the phenotype is restricted to the central retina.

Alterations affecting inner retinal neurons in the immediate stage accompanying photoreceptor death are known as phase 1 remodeling and mostly involve second order neurons (i.e. bipolar and horizontal cells) and retinal glia^18^. Because Tvrm4 mice of the present study are adult, we investigated whether the hallmarks of phase 1 remodeling observed in other forms of retinal degeneration (i.e. rd1 mice), in which phenotype onset overlaps to the late retinal synaptogenesis, also occur in the novel mutant. Morphological and functional data are reported in Supplementary Materials.

### Glial reactivity

**Figure 6:**
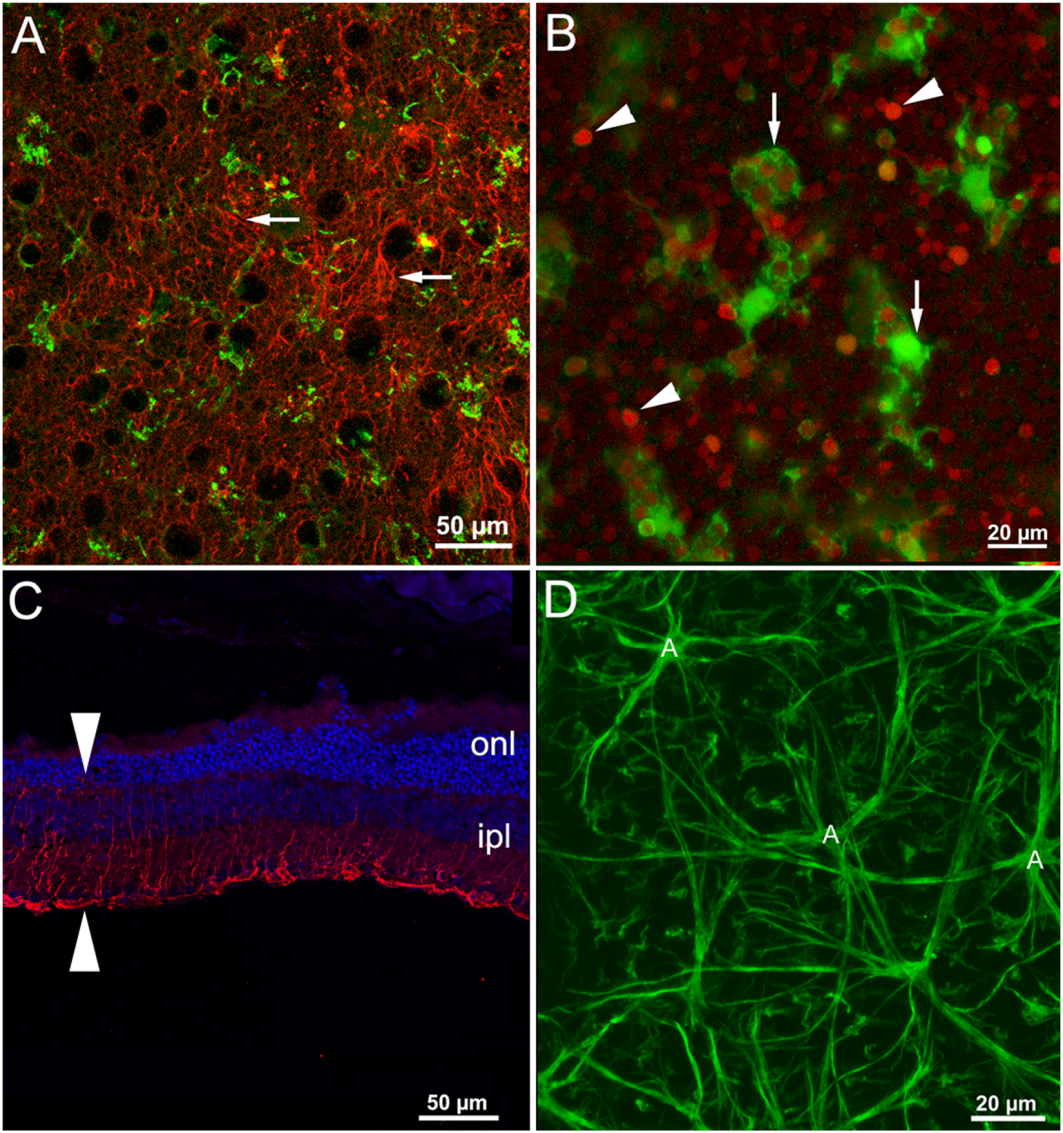
Glial activation. (**A**,**B**) Macro and microglia in the retina of Tvrm4 mice exposed to 12,000 lux for 1 min and examined after 2 days. (**A**) whole mount ICCH with Iba1 antibodies (green) labels activated microglial cells (arrows) in the outer retina. Red staining: GFAP. (**B**) Pycnotic nuclei of dying photoreceptors (ethidium staining, red) are observed in the ONL (arrowheads) and inside phagocytic microglial cells. (**C**) vertical section stained with antibodies against GFAP (red staining), which highlights the radial processes of activated Muller cells (arrows). Nuclei are stained blue with Hoechst. (**D**) whole mount retina of a Tvrm4 mouse, observed 7 days after exposured. GFAP staining shows hypertrophic processes of astrocytes (**A**) in the corresponding retinal layer, intermingled with Muller cell endfeet.

Microglia recruitment and macroglia activation accompany photoreceptor loss in the Tvrm4 mutant. Numerous microglial cells in active, amoeboid form are observed in the superficial part of the ONL since the very initial stage of photoreceptor death. Large (Iba1-positive) elements are seen to engulf the nuclei of dying rods (Fig. 6A). In the centralmost retina, hit by the brightest conditioning light, and almost everywhere in retinas exposed for times of 4′ and longer, large accumulations of membrane-bound DNA material can be observed in the ONL (i.e. Fig. 5). These formations stain intensely with fluorescent nuclear dyes and correspond to large apoptotic bodies originating from photoreceptors; they are constantly surrounded by activated microglia. Microglial cells in the inner retina, instead, largely maintain the quiescent form. Also, microglia is not activated outside the central area exposed to bright light.

Retinal macroglia also activates in parallel to photoreceptor death: as early as 48 hrs post-induction using 1 min, 12,000 lux exposure, Muller cells show the expected upregulation of GFAP immunoreactivity (Fig. 6C) throughout the light-stimulated area, but not spreading to the intact periphery. In vertical sections, radial processes of Muller cells show intense staining and increase in thickness, which become more evident at longer times post induction. Similarly, astrocytes, better visible in retinal whole mounts focusing on the ganglion cell layer, show increase in process thickness, elevated GFAP immunoreactivity as well as irregularity of the surface arrangement (Fig. 6D). Similar to Muller cells, alterations in astrocyte morphology are detected early after phenotype induction and are restricted to the central area of bright light exposure. However, when longer exposure times are used (i.e. 2′ and higher) the spread of microglial activation always exceeds the area of damaged photoreceptors.

### Retinal recovery

**Figure 7:**
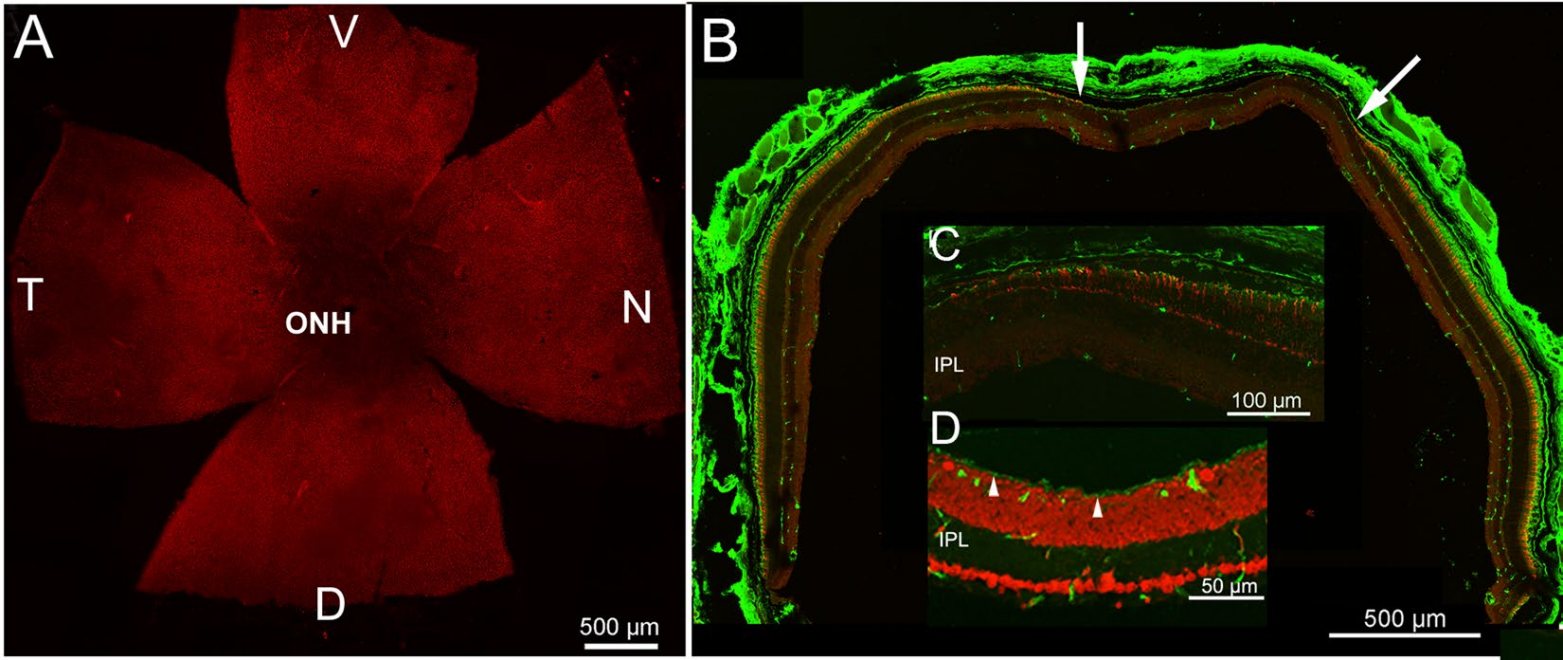
Long-term effects of phenotype induction. Whole mount retinas exposed for 1 min to 12,000 lux and examined after 30 days. (**A**) Focus on cone outer segments, stained red by cone opsin antibodies, show that the central, dark area, has decreased compared to what shown in Fig. 3A. ONH: optic nerve head. (**B**) Vertical section of an eye cup from a Tvrm4 mouse similar to that shown in A. Cone arrestin (red) and rhodopsin staining (green) show that the photoreceptor-free zone (between the white arrows) remained restricted to the central retina, similarly to what shown in Fig. 2A. (**C**) Cone progressive shortening toward the central retina (on the left side of the picture), where a faint rhodopsin staining becomes visible. (**D**) Light sensitive channel (green) highlights punctuate staining in the outer retina (arrowheads), likely corresponding to re-growing outer segments of rods. Red: ethidium nuclear counterstaining.

The analysis of the phenotype of Tvrm4 mice exposed for 1 min to 12,000 lux was followed up to 30 days after light-induction. At this time, microscopical analysis shows that the average size of the central degenerated zone did not expand compared to the average size measured at shorter times after induction (Fig. 7). One month post-exposure, the dark retinal area surrounding to the optic nerve head and emphasized by cone opsin staining had reduced to 21% of the whole retinal surface, compared to the 35% extension at 7 days and visible in Fig. 3A. Hence, phenotype worsening is restricted to the centralmost retinal zone and never spreads beyond the original boundaries, which actually become more limited.

**Figure 8:**
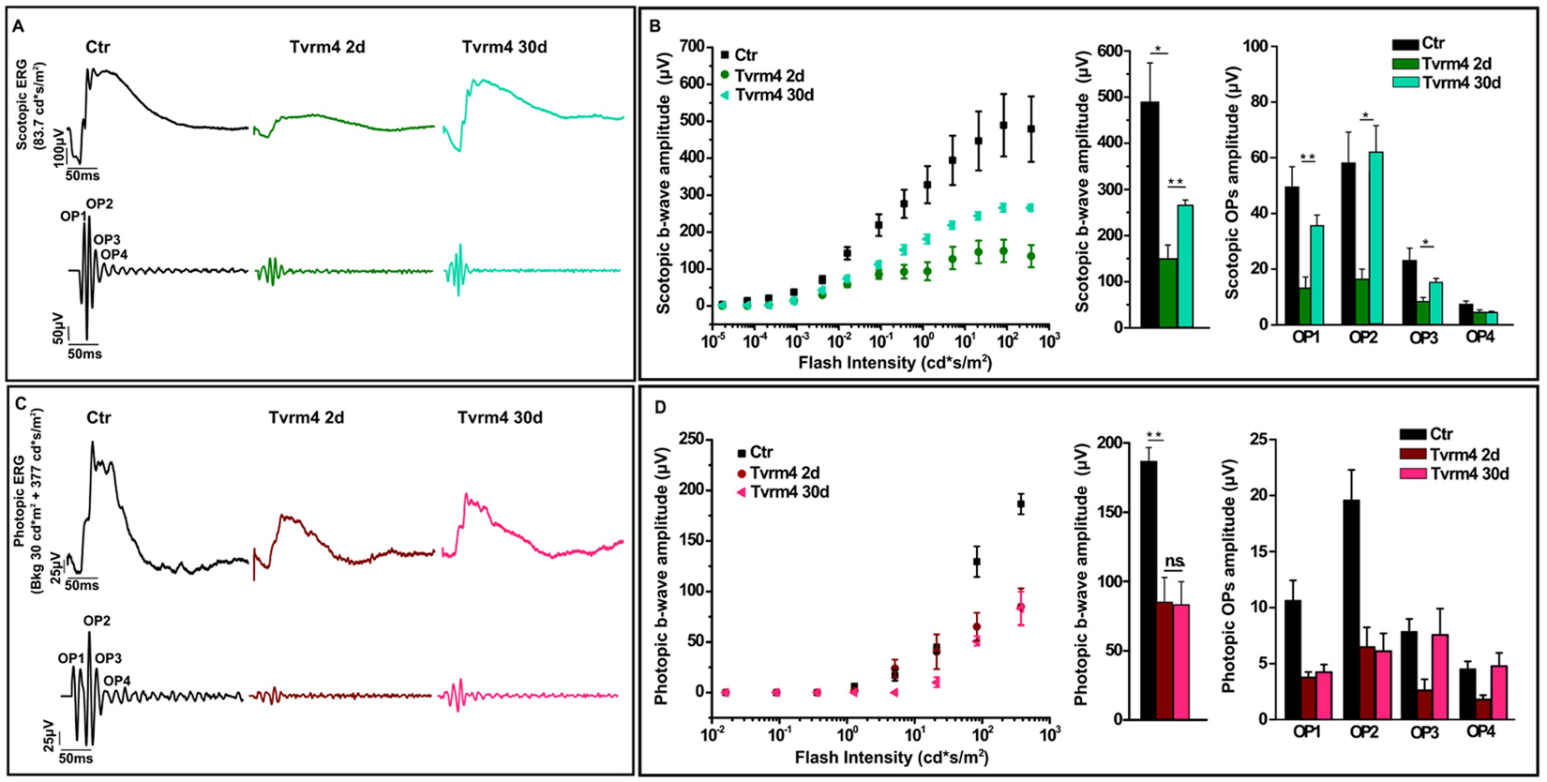
Recovery of retinal function 30 days post exposure. (**A**) Top traces: Representative scotopic flash ERG responses. Bottom traces: OPs filtered from the ERG response to the bright flash. (**B**) Left panel: scotopic b-wave amplitude as a function of flash intensity. Middle histograms: maximum b-wave amplitude measured in response to 83.7 cd*s/m^2^ from control and Tvrm4 mice exposed to 12,000 lux for 1 min and recorded 2 and 30 days after phenotype induction. Right panel: histograms of scotopic OPs amplitudes, showing a trend similar to the b-wave. (**C**) Top traces: representative photopic flash ERG responses. Bottom traces: OPs filtered from the ERG response to the brightest flash. (**D**) Left panel: photopic b-wave amplitude as a function of flash intensity. Middle histograms: maximum b-wave amplitude measured in response to 377 cd*s/m^2^. Right panel: histograms of photopic OPs amplitudes. N = 6 for each condition. Bars represent the mean ± SE *P < 0.05; **P < 0.01 one-way ANOVA.

Correspondingly, 30 days post induction, the amplitude of the scotopic ERG also partially recovers (Fig. 8). Figure 8A–C shows representative scotopic and photopic flash ERG waveforms and relative oscillatory potentials (OPs) responses from non-exposed Tvrm4 mice and from Tvrm4 mice recorded 2 and 30 days after light induction. The scotopic b-wave and OPs amplitude (OP1, OP2 and OP3) after 30 days are significantly increased compared to 2 days (Fig. 8B). To investigate where these functional changes originate, we analyzed the b-wave vs. a-wave ratio, which was found to remain constant at all time points analyzed (2 days: 4.2 ± 0.3; 30 days: 4.6 ± 0.6; ANOVA one-way, *P* = 0.63. Conversely, photopic ERG recordings show that the functionality of the cone pathway remained unvaried throughout the same period of time (Fig. 8D). The unexpected partial recovery of the scotopic ERG response is likely due to the particular rhodopsin mutation studied here, triggered by light acting directly on the protein, which then acquires toxic features for rods, leading to their death. However, rods located at the edge of the irradiated zone, escaping degeneration (either because not reached by enough inducing light or not irreversibly damaged) might clear toxic rhodopsin molecules, regenerate discs and recover. This is indicated physiologically by the recovery of the b/a ratio, as well as morphologically: immunostaining of retinal sections with light sensitive channel antibodies reveal short rod outer segments detected 30 days post exposure in an area flanking the central retinal “hole” (Fig. 7). This area latter is otherwise devoid of outer segments already 1 week post phenotype induction (Fig. 2).

In summary, Tvrm4 mutants offer the possibility to modulate extent and rate of photoreceptor degeneration by choosing an appropriate combination of light intensity and duration and portray an acute process of photoreceptor degeneration characteristic of human class II rhodopsin mutations.

## Discussion

This study provides the secondary characterization of retinal phenotype of Tvrm4 mice, which carry a dominant *RHO* mutation activated upon brief exposure to strong, white light. Tvrm4 mice, first devised developed in the laboratory of P. Nishina at Jackson’s^12^ offer the unique opportunity to trigger phenotype manifestation outside the period of retinal development, in young adulthood, mimicking the age of onset of typical human RP. This is very different from other, widely used retinal degeneration mice (i.e. rd1 and rd10 phosphodiesterase mutants) in which the mutation becomes expressed during the first 10 days of life, when photoreceptors undergo the final stages of maturation and synaptogenesis. The possibility to study photoreceptor death and neuronal remodeling bypassing the stages of retinal development and synapse formation is important because in humans, RP is a disease of adulthood, with symptoms that rarely overlap with retinal development. In addition, the overlay of photoreceptor degeneration and retinal development makes it difficult to distinguish between primary effects of the underlying mutation and retinal reorganization due to interactions of photoreceptor death and development completion^19–22^.

Here, we find that pattern of photoreceptor death, most relevant aspects of phase 1 remodeling, and corresponding ERG signature, are all very similar to those already described in rd1 and rd10 mice during the first month of life^19–22^ and to other mutants^23^, therefore we conclude that these are primary, pathognomonic signs of RP, independently from the age of onset of the phenotype and from the causative mutation.

Future studies comparing the results obtained here with a phenotype assessment in Tvrm4 immature mice will provide valuable information on the consequences of the Tvrm4 rhodopsin defect when combined with photoreceptor immaturity.

Unlike other *RHO* models of RP, Tvrm4 mice are not transgenics and therefore exhibit normal levels of rhodopsin. This is important in view of the notion that levels of rhodopsin in photoreceptors are tightly regulated and higher than normal amounts of this protein are believed to be toxic *per se* for the normal retina^24^. In Tvrm4 mutants, any abnormality observed upon light induction can be attributed to the sole effect of the mutation.

Tvrm4 mice exhibit the typical sensitivity to light of animals and humans with Retinitis Pigmentosa; numerous retinal degeneration mice have been shown to undergo faster retinal structural and functional decay if exposed to higher environmental light levels; conversely, photoreceptor degeneration slows down in dark rearing conditions^25^.

More specifically, Tvrm4 mice recall English Mastiff dogs with a T4R dominant mutation of *RHO* ^26, 27^, for their typical delay in dark adaptation and slow recovery after photobleaching, described for Tvrm4 mutants in the original paper by^12^. Together with normal retinal structure and rhodopsin expression, defects in dark adaptation and a highly localized pattern of initiation of photoreceptor degeneration are hallmarks of Tvrm4 mutants, English Mastiff dogs and RP patients with so-called class B1 *RHO* mutations^28^. Noticeably, T4R dogs are extremely sensitive to environmental light, and even moderate illumination levels, as used in routine ophthalmological examination, accelerate retinal degeneration.

As for the T4R dogs, we used heterozygous Tvrm4 mice for our study, to better mimic the genetics of dominant human mutations of Rho; however, the homozygous individuals we examined did not show any visible difference from the heterozygous in terms of severity of the induced phenotype and pattern of retinal degeneration.

The phenotype induced in Tvrm4 mice by strong light is likely triggered by accumulation of large amounts of bleached mutant rhodopsin, which shows a gain of function ultimately leading to photoreceptor death. We made no attempts to trace the cellular pathways connecting the Tvrm4 *RHO* mutation to rod demise but this is indeed an important topic to study, given the relevance that *RHO* genetic defects play in human RP.

In these studies, one could also investigate the very interesting topic of non-cell-autonomous signaling among retinal cells. Noteworthy is the fact that the death signals involving i.e. photoreceptors, activated macro and microglia, blood vessels, located at the interface between illuminated and dark retinal areas, remain confined to a central spot and do not affect the survival of adjacent cells. The Tvrm4 pattern of degeneration is a typical rod-cone decay in which non-cell-autonomous mechanisms of survival and death (like the interdependence of cones from rods^29, 30^) can be adequately studied; the adjacent, virtually intact retinal area can serve an internal control for a number of functional, biochemical and molecular biology investigations.

Most likely, partially damaged rods at the border between severely damaged and intact area can recover, possibly when the bleached rhodopsin accumulated in the outer segments has been cleared and new discs, with freshly synthetized protein, are generated at the outer segment base. The process of synthesis of new discs is known to take place at a rate of about 2 µm/day in mouse rods^31^, so that entire outer segment renewal occurs in a time span of 2–3 weeks: this would explain the reduction of the irradiated zone measured after this period, the increased staining with rod outer segment markers and the recovery of the a-wave of the ERG observed thereafter. Thus, Tvrm4 mutants provide unique models (also exploitable for ***in vitro*** studies) to test accurately light stimuli acting as irreversible or reversible conditions for rod survival and outer segment genesis in an adult mammalian with a rhodopsin dominant mutation, thus opening the path to cell biology studies of great value for photoreceptor biology and repair.

A further advantage of the model is represented by the possibility of exposing to the inducing light one eye only. Dilating only one pupil completely prevents induction of the phenotype in the other retina, thus providing a useful control when simultaneous testing of the two eyes is desirable. Examples include monocular gene therapy^32^, monocular pharmacological treatments followed by morphological and ERG assays^33^, retinal/specific gene expression, studies of the effects of photoreceptor degeneration on cortical plasticity^34^ etc.

In view of the very different areas that are illuminated with different exposing protocols, we conclude that a) short time exposure are recommended for morphological studies in which wild type, non-induced areas can be unequivocally identified by photoreceptor staining methods; these protocols are very useful when cell interactions occurring at the interface between intact and degenerating tissues are being studied. An example is represented by local factors affecting the survival of rods and cones. b) medium time exposures (2 minutes) are optimal for the combination of morphological, biochemical and mass-electrophysiology responses and for possible rescue studies; c) long time exposures (5 minutes), at lower intensity, are ideal for ablating a large population of photoreceptors simultaneously and to study structural and functional effects on the inner retina (morphological and functional remodeling), for instance using electrical imaging methods to reveal the activity of bipolar cells^35^.

When a short time exposure (typically, 1 minute) is used, a nasal-temporal asymmetry in the pattern of degeneration may occur in some animals. This has to be taken into account during the experimental design; however, the effective pattern of induction can be visualized easily in retinal whole mounts stained post-mortem.

Overall, the degeneration pattern triggered in the Tvrm4 is rather acute (first effects detected after 2 days, largely completed in 2 weeks) but still manageable for performing structural and functional analysis; moreover, the spatial extent of retinal degeneration can be modulated by changing the illumination conditions, i.e. longer time exposures at lower intensities lead to larger areas of retinal degenerations. This is likely due to the fact that the mouse has a highly-curved eye, which makes it difficult to illuminate the retina completely, in a very short time, in awake and moving animals. Longer time exposures increase the probability of the light to reach and trigger the phenotype in peripheral zones of the eye; this experimental paradigm might be chosen when recording responses from large retinal areas (i.e. using multielectrode arrays^36^; or calcium imaging^37^). Compared to commonly used phosphodiesterase mutants, Tvrm4 mice recall rd10 mice insofar complete photoreceptor degeneration occurs within about one month from phenotype onset^19^ making functional studies possible; yet, the initial phase of rod death is quite fast in the rhodopsin mutant studied here and more reminiscent of photoreceptor death onset in the aggressive rd1 phenotype^22^. However, the Tvrm4 degeneration turns into retinal degeneration only as a consequence of interaction of light and rhodopsin and the phenotype might affect only part of the retina. This explains the persistency of an ERG b wave even at the latest times studied here, unlike what observed in rd1 and rd10 mutants, in which the ERG extinguishes after about 2 and 9 weeks of life, when rods and cones have degenerated completely.

Altogether, Tvrm4 mutants provide excellent opportunities to study dominant RP and can be used to unravel mechanisms and pathways of cell death associated to numerous mutations of *RHO* as well as to test rescue strategies for a common cause of the human disease.

## Methods

### Mouse lines and animal used

Animals were treated in accordance to Italian and European institutional guidelines, following experimental protocols approved by the Italian Ministry of Health (Protocol #14/D-2014, CNR Neuroscience Institute; Protocol #DGSAF0001996/2014, Department of Pharmacy, University of Pisa) and by the Ethical Committees of both Institutions. Protocols adhere to the Association for Research in Vision and Ophthalmology (ARVO) statement for the use of animals in research.

Heterozygous (Ht) Tvrm4 mice (Rho^Tvrm4^/Rho^+^) with a I307N (near C-terminus) mutation of the rhodopsin gene (*Rho*) and C57Bl6J wild type (wt, littermates) mice were used for this study^12^. Tvrm4 mutants are on a C57Bl6/J background and exhibit no retinal phenotype unless exposed to bright light stimuli. Genotyping was performed following^12^ and Jackson laboratories indications.

Seven wt and 27 Ht Tvrm4 mice were used to assess retinal histology after 1 minute (min), 12,000 lux exposure; they were examined at 48 hr, 7,14, 21 and 30 days post induction, using for a minimum of 3 and a maximum of 8 Ht mice per group.

Fourteen additional Ht Tvrm4 mice were used for retinal histology following 2 min, 12,000 lux exposure. A minimum of 3 and a maximum of 5 mice per group were used for studies at 48 hr, 7, 14 and 21 days post induction. N = 3 animals were used for histological studies at 48 hr post induction; n = 8 mice for studies after 7 days. A total of 5 wt mice were used for controls.

A total of 3 Ht and 2 wt mice were used for retinal histology after exposure to 12,000 lux inducing light for 5 min and examined 7 days post exposure. Finally, 9 mice were used to study the effects of 6,000 lux exposure for 3, 5, and 10 min. Mice were 3 per group; those exposed for 3 min were harvested after 48 hr; those exposed for 5 and 10 min were harvested after 7 days. In total, 64 Tvrm4 Ht and 14 wt mice were used for histology.

Additional homozygous (n = 3) and Ht (n = 6) Tvrm4 mice were used for initial experiments to set up illumination conditions, duration of the inducing light and demonstration of the possibility of inducing unilateral retinal degeneration by dilating only one pupil.

Sixty additional Ht Tvrm4 mice (at least n = 3 for experimental group) and 6 wt (littermates) were used for ERG recordings after exposure to different inducing light intensities and assessment after various times, as detailed below.

### Induction of retinal degenerating phenotype

Ht Tvrm4 mice and wt littermates (all aged 2–5 months) were dark adapted for 4 hr, then given 1 µl eye drops of 0.5% atropine (Allergan); after 10 min, mice were placed in a black box (140 × 30 cm size; 30 cm height) lined with mirrors on the inner sides and closed with a lid holding 3 neon bulbs (Philips Master TL5 HE 28 W/840, length 115 cm; 16 cm in diameter; Cool White mercury lamps). Light intensity could be varied by changing the number of illuminated bulbs and was monitored by inserting in the box a light meter sensor (mod. LX103, Lutron, Digital Instruments); duration of exposure could be programmed with a digital clock controlling the light bulb switch. Mice were exposed to light pulses of 6,000 or 12,000 lux having durations of 1, 2, 3 or 5 minutes. In the initial experiments, during the light pulses, the temperature of the cage in the illuminating box was monitored with the precision of 1/10 °C using a digital thermometer whose sensor was placed inside the box. No changes were observed with respect to identical measurements done in the absence of the light pulses. Most of the phenotypical analysis refers to a single light exposure of 1 min having an intensity of 12,000 lux. Control animals were a) wild type (wt) littermates of Tvrm4 mice, also exposed to bright light pulses of identical intensity and duration; b) Ht Tvrm4 mice which were not administered atropine; c) Ht Tvrm4 mice in which only one eye was treated with atropine. After bright light exposure, mice were returned to their cages and maintained in a local facility with water and food *ad libitum* on a 12/12-hour light/dark cycle, with illumination levels below 60 lux.

To verify the possibility of induction of anesthetized animals, 3 Tvrm4 mice were anesthetized with Avertine as above, pupil-dilated, placed in a stereotaxic apparatus, maintaining the animals immobilized and with their eyelids open, and exposed for 2′ at 12.000 lux. One awake, non-restrained Tvrm4 mouse was also exposed to the same inducing protocol. ERG recording was performed after 48 hours on these 4 mice. Only the awake Tvrm4 mutant did develop a retinal degeneration phenotype, while the anesthetized animals remained unaffected, returning minimal ERG decrements. We concluded that an effective induction protocol requires the presence of intact eye movements, probably necessary to maximize the retinal illuminated (and thus degenerated) area. Eye movements are greatly reduced by most anesthetics including Avertine^38, 39^.

### Tissue preparation, histology and immunocytochemistry (ICCH)

For retinal histological studies, 2, 7, 14, 21 and 30 days after light induction, mice were deeply anesthetized with intraperitoneal injections of 0.1 ml/5 g body weight Avertin (3-bromo-ethanol in 1% tert-amyl alcohol), their eyes quickly enucleated and the animals killed by cervical dislocation. Eyes were labeled on the dorsal pole, the anterior segments removed to obtain eye cups and fixed in 4% paraformaldehyde (PFA) in 0.1 M phosphate buffer, PB, pH7.4, for 30′ or 1 hour, at room temperature. Eye cups were washed extensively in PB, infiltrated in 30% sucrose in PB, frozen in Tissue-Tek O.C.T. compound (4583, Sakura Olympus, Italy) using cold isopenthane and stored at −80 °C until use. Additional whole eyes were also stored using the same protocol. Consecutive cryostat sections (12–14 µm thick) were collected on Super Frost slides and used for immunocytochemistry (ICCH).

Contra-lateral eyes (or eyes from different mice) were used to prepare retinal whole mounts, in which the retina was separated from the pigment epithelium and flattened by making four radial cuts toward the head of the optic nerve, maintaining a reference on the dorsal pole. ICCH on both retinal sections and whole mounts was performed following^40^, by incubation in a) block solution with 0.3% Triton-X 100, 5% of the serum of the species in which the secondary antibody was generated and 0.01 M Phosphate Buffer Saline (PBS); incubation time was 2 hr for the sections and overnight for whole mounts; b) primary antibody (Ab), diluted in PBS, 0.1% Triton-X 100 and 1% serum; incubation time was overnight for the sections and 3 days for whole mounts; c) fluorescent secondary Ab, diluted as the primary Ab; incubation time was 2–3 hr for the sections and 2 days for whole mounts. Incubations steps longer than 2 hr were done at 4 °C.

Mouse monoclonal, primary Abs used for retinal sections were: Rhodopsin (O4886 Sigma-Aldrich, Italy; diluted 1:1000); PSD95 (13552 AbCam, Cambridge UK; diluted 1:500); synaptotagmin 2 (Znp1 Zebrafish International Resource Center, USA; diluted 1:800); alpha G0 (MAB3073 Chemicon, Merck-Millipore, Italy; diluted 1:800); light sensitive channel (kindly donated by Robert Molday, University of British Columbia, Vancouver, Canada), used 1:1000); non-Phosphorylated Neurofilament H (SMI32R, Covance, CA, USA; diluted 1:800); Protein Kinase Cα (PKCα; P5704, Sigma-Aldrich, Italy; diluted 1:800).

Rabbit polyclonal, primary Abs were: S and M/L cone opsins (AB5405, AB5407; Merck-Millipore, Italy, diluted 1:800); cone arrestin (AB15282, Merck-Millipore, Italy; diluted 1:5000); Calbindin D (CB38a, Swant Ltd., Switzerland, diluted 1:500); Protein Kinase Cα (PKCα; P4334, Sigma-Aldrich, Italy; diluted 1:800); Glial Fibrillary Acidic Protein (GFAP; G9269, Sigma-Aldrich, Italy; diluted 1:1000). A sheep mGluR6 polyclonal antibody was a kind gift of Catherine Morgans (Oregon Health and Science University, Portland, OR USA; diluted 1:200). A goat Choline Acetyltransferase (AB144P, Merck-Millipore, Italy; 1:500) polyclonal was also used. Abs against cone opsins, calbindin D, Iba1 (019-19741, Wako, USA; diluted 1:500), Neurofilament 200 kDa (N0142, Sigma-Aldrich, Italy; diluted 1:500) and PKCα were also used in whole mount preparations, at 10x the concentrations used for sections.

Secondary antibodies were: donkey anti-mouse Alexa Fluor 488 (A-21202, Life Technologies, Italy); donkey anti-rabbit Rhodamine Red X (715296151); donkey anti-goat (705-546-147); and Donkey Anti- Sheep (713-546-147), all from Jackson ImmunoResearch laboratories, USA; these were diluted 1:1,000 for retinal sections and for whole mount preparations. For nuclear counterstaining, retinal sections were incubated for 2 minutes in Hoechst (33342) or in Ethidium homodimer-1 (E1169), both from Life technologies, Italy, diluted 1:1,000. After rinsing in PBS, specimens were mounted in Vectashield (H-1000; Vector Laboratories, Burlingame, CA) and coverslipped.

Images of retinal preparations were obtained with a Zeiss Imager.Z2 microscope equipped with an Apotome2 device (Zeiss, Milan, Italy), using a Plan Neofluar 40x/1.25 oil objective; or with a Leica TCS-SL confocal microscope equipped with 488 and 453 lasers, using a Plan Apochromat 40x/1.40 oil objective. Images were saved as tiff files; brightness and contrast were adjusted with the Zeiss software ZEN®PRO 2012 or with Adobe Photoshop. Retinal whole mounts were also imaged with the Imager.Z2 microscope using EC Plan-Neofluar 5x/0.16 M27, 10x/0.3 M27 and 20x/0.50 M27 objectives; images were tiled with ZEN module “Tiles & Positions” software to reconstruct the entire retinal surface.

Areas of maximum photoreceptor loss were measured on retinal whole mounts stained for cone specific opsins and mounted photoreceptor side-up. Full retinal montages were obtained with Apotome2 using a 20x objective, saved as tiff files and transferred to a Metamorph image analyzer. Images were homogeneously thresholded for brightness and the darkest, central region, surrounding the optic nerve head (ONH), automatically traced and measured. The diameter of the photoreceptor-degenerating zone was also measured on equatorial retinal sections stained with photoreceptor specific antibodies and nuclear dies. Retinal vertical sections (n = 3 per retina, 3 retinas per time point) were used to measure the ONL thickness 48 hr, 7, 14 and 30 days after light exposure. A 20x objective and a Zeiss Axiocam color camera were used to take an image per section, centered to the ONH and measuring 600 × 400 micrometers. Rows of nuclei in the ONL were counted semi-manually with Metamorph and the data averaged.

### Electroretinogram (ERG) recordings

Animals were anesthetized with 20% Urethane (Sigma Aldrich, Milan, Italy), used at a concentration of 0.1 ml/10 g body weight. ERGs were recorded from dark-adapted mice by means of coiled gold electrodes making contact with the cornea moisturized by a thin layer of gel. Pupils were fully dilated by application of a drop of 1% atropine (Farmigea, Pisa, Italy). Light stimulation and data analysis were as previously described in detail^41, 42^. Scotopic ERG recordings were average responses (n = 5) to flashes of increasing intensity (2.19*10^−4^ to 83.7 cd*s/m^2^, 0.6 log units steps) presented with an inter-stimulus interval ranging from 20 s for dim flashes to 1 min for the brightest flashes. Isolated cone (photopic) components were obtained by superimposing the test flashes (0.016 to 377 cd*s/m^2^) on a steady background of saturating intensity for rods (30 cd/m^2^), after at least 15 min from background onset. Amplitude of the a-wave was measured at 7 ms after the onset of light stimulus and the b-wave was measured from the peak of the a-wave to the peak of the b-wave. Oscillatory potentials (OPs) were also measured in both scotopic and photopic conditions. The b wave OPs were extracted digitally by using a fifth-order Butterworth filter as described before^43, 44^. Peak amplitude of each OP (OP1–OP4) was measured. The ERG data for each condition of light-induction were collected from at least 6 different animals.

## Electronic supplementary material

Gargini et al. supplementary
