# Supplementary material for: Pattern of retinal morphological and functional decay in a light-inducible, rhodopsin mutant mouse
Source: Sci Rep. 2017 Jul 18;7:5730. doi: 10.1038/s41598-017-06045-x (PMC5516022; doi:10.1038/s41598-017-06045-x)
Supplement: Supplementary file 1 — Gargini et al. supplementary [file 41598_2017_6045_MOESM1_ESM.pdf]

Supplementary Information

Pattern of retinal morphological and functional decay in a light-inducible, rhodopsin mutant mouse

Claudia Gargini, Elena Novelli, Ilaria Piano, Martina Biagioni, Enrica Strettoi

Inner retinal morphological and functional changes

Tvrm4 mutants mimic faithfully dominant RP and can be used to study mechanisms and pathways of cell death associated to mutations of *RHO* as well as the occurrence of inner retinal remodeling. Because Tvrm4 mice of the present study are adult, we investigated whether the hallmarks of phase 1 remodeling observed in other forms of retinal degeneration (i.e. rd1 mice), in which phenotype onset overlaps to the late retinal synaptogenesis, also occur in the novel mutant.

We studied the morphology of the dendrites of bipolar and horizontal cells in the OPL and examined the morphology of the innermost retinal neurons in Tvrm4 mutants exposed to 12,000 lux for 1 min after 7, 14 and 21 days. We observed a significant shedding of dendritic arborization from rod bipolar cells, topographically associated with the process of rod degeneration (Figures S1, S2). Dendritic retraction is paralleled by decreased expression and mislocalization of mGluR6 receptors in rod bipolar cells; mGluR6 immunoreactive puncta are reduced in number and become misplaced along the axons of rod bipolar cells in the IPL (Figure S2B). Dendritic retraction is clearly detectable 7 days post induction at retinal locations with prominent rod loss and ONL rows lower than 6. mGluR6 decrement and misplacement is an early indicator of OPL abnormality in the central retina, detectable 48 hours post light induction.

In parallel to rod bipolar remodeling, horizontal cells begin to show hypertrophic bodies; both dendrites and axonal endings exhibit major discontinuities in their spatial arrangement and decrement in the complexity of their process network, clearly perceivable in retinal whole mounts (Figures S1C, D and S2E, F). Particularly evident are the changes of axonal arborizations (postsynaptic to rod terminals), which decrease in density but become thicker and spatially irregular. These abnormalities occur inside the area irradiated by the inducing light and match the zone of photoreceptor death. Sprouting of second order neurons, described in other mutants, is virtually absent here and regressive events in the outer retina predominate. As for other rodent models, changes in specific types of cone bipolar cells are not easily detectable, for specific markers are rare. A thinning of the OPL in the retina of Tvrm4-induced mice may be appreciated when dendrites of second order neurons are stained with specific antibodies (Figure S2C, D). The innermost retina, however, does not show major signs of morphological abnormalities in the neuronal components. Cholinergic amacrine cells, a landmark of retinal laminar organization, and neurons in the ganglion cell layer show no obvious sign of remodeling (Figure S2E, F). In the central retina, regression of rod bipolar and

horizontal cells becomes more severe with time and their dendritic compartments are completely lost within 3 weeks post induction. After 4 weeks, the central retina is only composed by the inner layers only and the OPL is no more distinguishable (not shown). A parallel, follow up study of Tvrm4 mice is in progress to specifically characterize remodeling and to estimate inner neuronal survival in this mouse model.

Information on the functional properties of inner retinal cells in both scotopic and photopic conditions was obtained by analyzing the oscillatory potentials (OPs) of the ERG. A significant amplitude reduction of scotopic OP1 ($p=0.005$, t-test), OP2 ($p=0.021$, t-test) and OP3 ($p=0.027$, t-test) is observed as early as 2 days after exposure to 12,000 lux for 1 min (Figure S3A and C). Conversely, the photopic OPs (OP1, OP2 and OP3) are not significantly affected (Figure S3B and D). These observations confirm the effects described above on the main ERG components and support the notion that in this animal model the primary damage starts in the rod pathway and eventually involves cones.

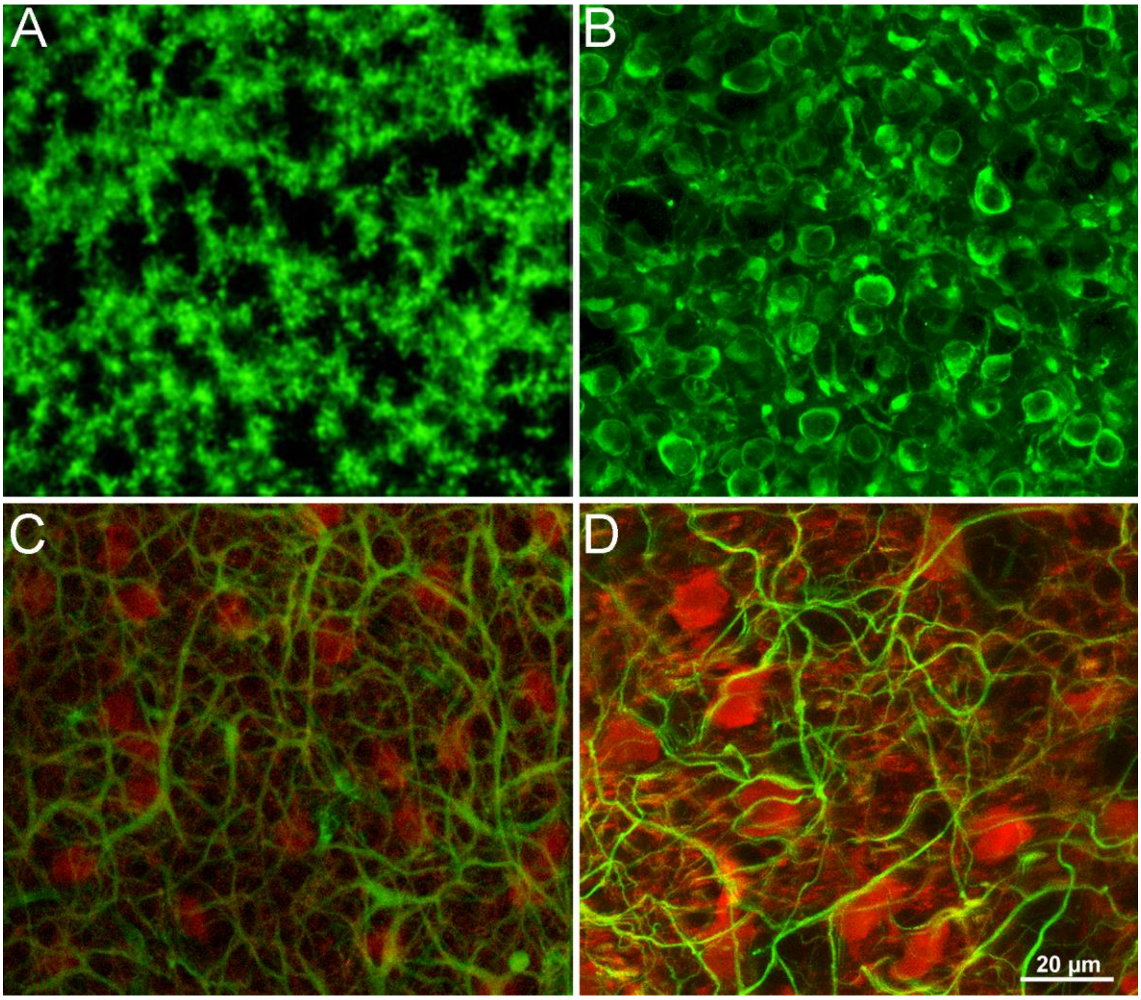

Figure S1. Inner retinal remodeling: rod bipolar and horizontal cells

Dendrites of rod bipolar cells stained by PKC antibodies in whole mount retinal preparations from a control (not-induced Tvmr4) mouse (A) and a Tvrm4 mouse exposed for 1 min to 12,000 lux and observed after 7 days (B). Focus is on the OPL, which shows profusely ramified dendrites in A but cell bodies with bare and aberrant dendrites in B. C, D: same preparations as above, stained for Calbindin D (red) and neurofilament (green), showing cell bodies/dendrites and axonal compartments of horizontal cells, respectively. Note the hypertrophic somas of horizontal cells and the loose and highly irregular meshwork of their axonal arbors.

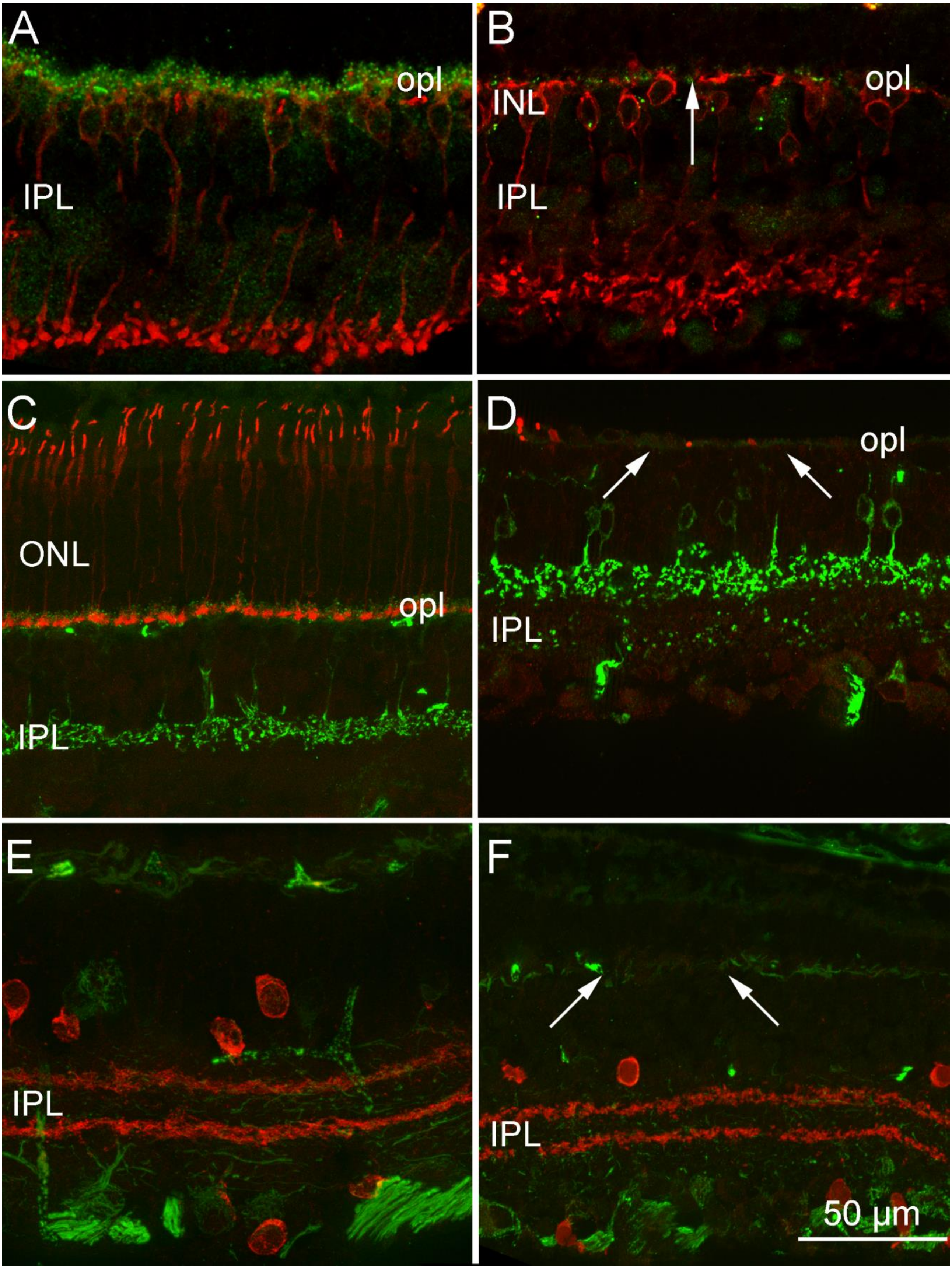

Figure S2. Inner retinal remodeling: bipolar, amacrine and ganglion cells

The inner retina of a control (not-induced Tvmr4) mouse (A, C, E) and a Tvrm4 mouse (B, D, F) exposed for 1 min at 12,000 lux and examined after 14 days. A, B: PKC staining (red) of rod bipolar cells highlights dendritic retraction in B. mGluR6 positive puncta (green) decorate profusely the OPL in A but are rare

(arrow) and misplaced in the INL in B. C, D: Cone arrestin staining (red) underlines disappearance of cones from the central retina of Tvrm4, light induced mice (D). Synaptotagmin staining (green) shows that dendrites of cone bipolar cells and horizontal cells labeled with this antibody are no more visible in the OPL (arrows), while axons of cone bipolars are still intact in the IPL. E, F: ChAT staining of cholinergic amacrines (red) and neurofilaments (green) demonstrate a relatively normal morphology of the innermost retinal layers in Tvrm4 mutants (F). Arrows in F point to the lack of neurofilament positive processes in the OPL, due to horizontal cell regression.

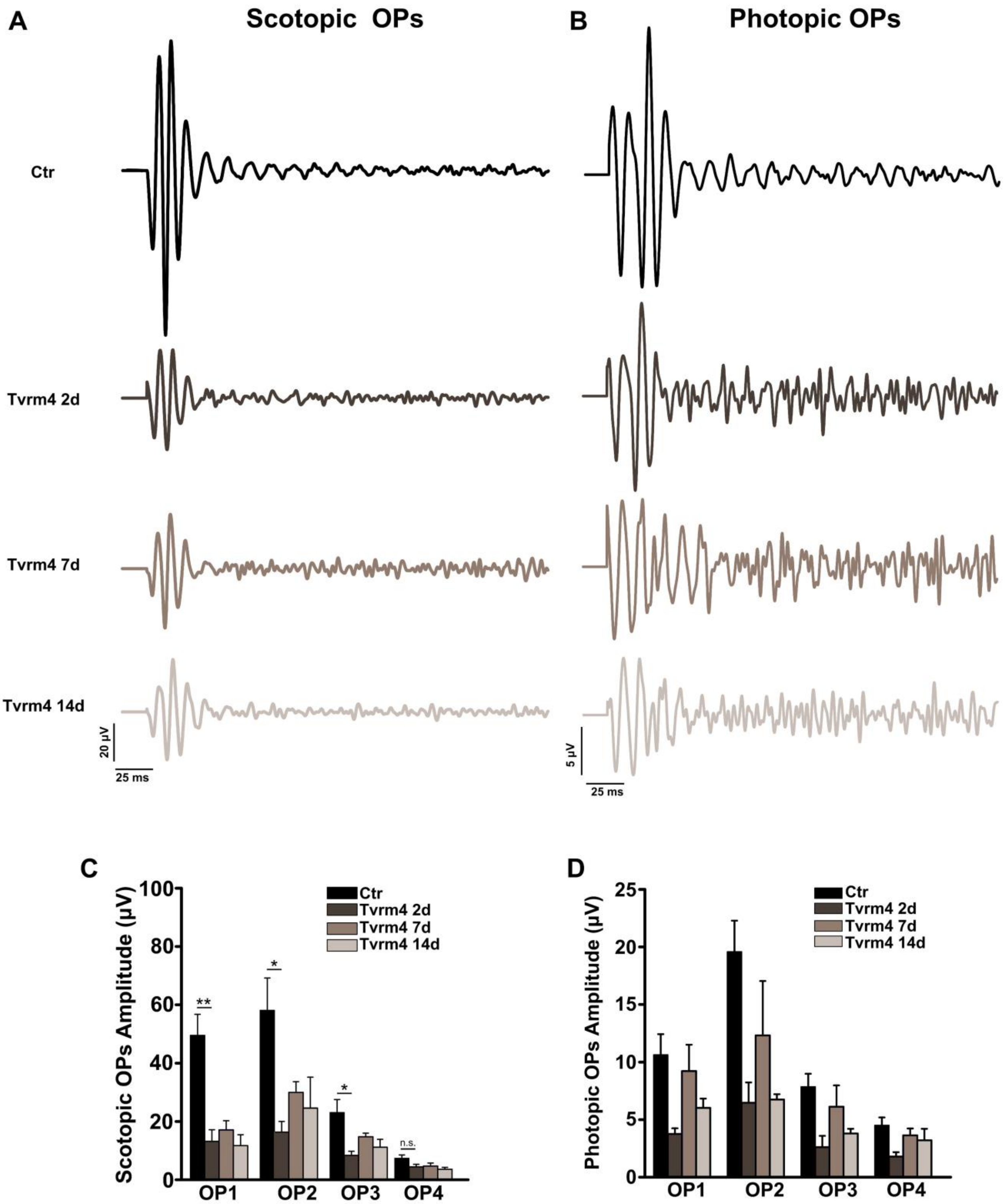

Figure S3. Functional decay at different times after light induction

Exposure to 1 min conditioning light of 12,000 lux , examination after 2, 7 or 14 days. (A,B) Representative examples of OPs obtained from scotopic and photopic ERGs, respectively. (C, D) Average amplitude of oscillatory potentials (OP1 - OP4) extracted from scotopic and photopic ERG responses to the brightest flash (377 cd*s/m^2. The amplitude of OPs is significantly reduced in Tvrm4 mice exposed to steady light with respect to control animals. OPs signals are impaired as early as 2 days from phenotype induction. n=6 for each condition. Bars represent mean ± SE * P<0.05; ** P<0.01 one-way ANOVA.
